# Flap fixation reduces seroma in patients undergoing mastectomy: a significant implication for clinical practice

**DOI:** 10.1186/s12957-016-0830-8

**Published:** 2016-03-08

**Authors:** James van Bastelaar, Arianne Beckers, Maarten Snoeijs, Geerard Beets, Yvonne Vissers

**Affiliations:** Department of Surgery, Atrium Medical Center Heerlen, Heerlen, The Netherlands; Department of Surgery, Orbis Medical Center Sittard, Sittard, The Netherlands; Department of Surgery, Maastricht University Medical Center, Maastricht, The Netherlands

## Abstract

**Background:**

Seroma formation is a common complication following mastectomy for invasive breast cancer. Mastectomy flap fixation is achieved by reducing dead space volume using interrupted subcutaneous sutures.

**Methods:**

All patients undergoing mastectomy due to invasive breast cancer or ductal carcinoma in situ (DCIS) were eligible for inclusion. From May 2012 to March 2013, all patients undergoing mastectomy in two hospitals were treated using flap fixation. The skin flaps were sutured on to the pectoral muscle using polyfilament absorbable sutures. The data was retrospectively analysed and compared to a historical control group that was not treated using flap fixation (May 2011 to March 2012).

**Results:**

One hundred and eighty patients were included: 92 in the flap fixation group (FF) and 88 in the historical control group (HC). A total of 33/92 (35.9 %) patients developed seroma in the group that underwent flap fixation; 52/88 (59.1 %) patients developed seroma in the HC group (*p* = 0.002). Seroma aspiration was performed in 14/92 (15.2 %) patients in the FF group as opposed to 38/88 (43.2 %) patients in the HC group (*p* < 0.001).

**Conclusions:**

Flap fixation is an effective surgical technique in reducing dead space and therefore seroma formation and seroma aspirations in patients undergoing mastectomy for invasive breast cancer or DCIS.

## Background

Seroma formation, a collection of serous fluid that contains blood plasma and/or lymph fluid, is a common side effect after mastectomy. It has an incidence of 3 to 85 % [1], and some surgeons regard seroma formation as an inherent part of breast cancer surgery. Seroma formation and its sequelae form the mainstay of complications in breast cancer surgery, varying from delayed wound healing, infection, skin flap necrosis and patient discomfort [2]. Patient discomfort seems mainly to be caused by frequent seroma aspirations and repeated visits to the outpatient clinic to deal with seroma and its sequelae.

Seroma formation continues to be a problem for patients undergoing surgery of the breast and/or axilla for invasive breast cancer. Pathogenesis of seroma is not yet fully understood. Several factors are held responsible for seroma formation. In a retrospective cross-sectional study by Hashemi et al., patients undergoing modified radical mastectomy (MRM) were shown to be 2.5 times more likely to develop seroma [3]. Gonzalez et al. analysed patients undergoing wide local excision and MRM for invasive breast cancer and concluded that the type of operation performed was the only predictor of seroma formation [4], whereas age of the patient, the presence and number of positive axillary lymph nodes, the total number of axillary lymph nodes removed, tumour size, weight of the patient and the use of neoadjuvant chemotherapy were not. The use of electrocautery has been demonstrated to increase seroma formation following mastectomy [5]; however, no other surgical devices (laser scalpel, argon diathermy and ultrasonic scalpel) or substances have proven to be superior in seroma reduction [6].

Seroma formation after axillary dissection for breast cancer cannot be avoided, but it can possibly be minimised by mechanical closure of the dead space [7]. Prospective trials have demonstrated that flap anchoring after mastectomy, and therefore dead space reduction, could be very beneficial [8]. The trial performed by Almond et al. on flap fixation compared to closed suction drainage showed no difference in seroma rates, but patients without drains were discharged earlier [8]. In the trial performed by Sakkary, the amount of fluid drained was significantly less in the flap fixation group [9]. In these studies, however, patient discomfort in terms of seroma aspiration and repeated hospital visits were not assessed. The most recently published retrospective study on quilting of the skin flaps after mastectomy and/or axillary lymph node dissection showed promising results [10]. In this study, quilting of the skin flaps led to less seroma formation, fewer seroma aspirations and fewer surgical site infections (SSIs).

A systematic review performed by Kyeong-Tae Lee et al. [11] in seroma formation after latissimus dorsi muscle harvesting suggests that dead space formation is one of the leading mechanisms in seroma formation. Prolonged leakage into the dead space by disrupted lymphatics and blood vessels most likely contributes to seroma formation [11]. The pathogenesis of seroma formation and its contributing mechanisms are not yet fully understood and require further investigation.

We hypothesise that obliteration of the dead space using sutured flap fixation following mastectomy will significantly reduce seroma formation, resulting in fewer seroma aspirations and outpatient visits. The aim of this retrospective observational cohort study is to demonstrate that patients undergoing mastectomy with flap fixation in combination with low suction drainage undergo fewer seroma aspirations.

## Methods

This retrospective study was conducted in the breast units of two large hospitals in the Netherlands (Atrium Medical Centre, Heerlen and Orbis Medical Centre, Sittard). The hospitals’ joint medical ethical committee granted approval (13-N-77), and informed consent was waived.

All patients undergoing mastectomy, mastectomy and sentinel node procedure or modified radical mastectomy for invasive breast cancer or ductal carcinoma in situ (DCIS) older than 18 years of age were eligible for inclusion. Patients undergoing direct breast reconstruction were excluded from this study. All patients received 2 g of cefazolin prophylaxis preoperatively. Low vacuum drains were inserted in all patients before wound closure. Drains were removed if fluid production was less than 50 mL/24 h. After 7 days, all drains were removed, irrespective of drain output. Five dedicated breast cancer surgeons performed the procedures. Patients were analysed in two separate 10-month intervals in which the method of wound closure differed.

### Historical control group (HC)

From May 2011 to March 2012, patients underwent conventional wound closure using subcutaneous and intradermal absorbable sutures. Closed suction drainage was applied to all patients.

### Flap fixation group (FF)

**Fig. 1:**
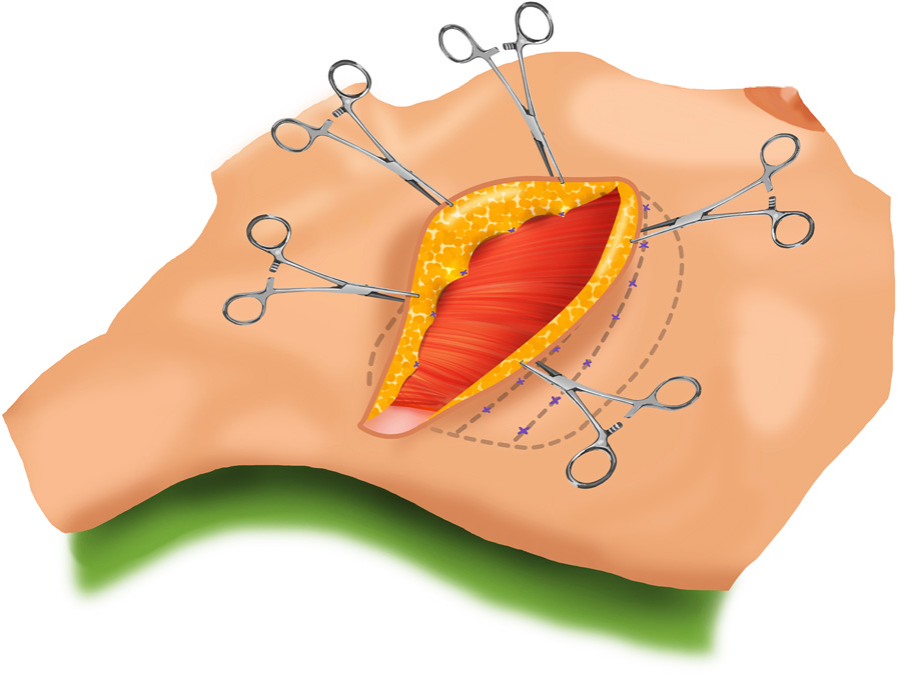
Schematic depiction of points of flap fixation

From May 2012 to March 2013, all patients underwent wound closure using flap fixation. After the mastectomy, the skin flaps were sutured on to the pectoral muscle with polyfilament absorbable sutures (Vicryl 3.0). Sutures were placed at 3-cm intervals in two or three rows, depending on the extent of the skin flaps (Fig. 1). This procedure was done as described by Almond et al. in 2010 [8], without closing the axillary dead space with sutures. Care was taken to prevent dimpling of the skin.

Data was extracted from electronic patient files by one of the co-authors. Operation reports and physicians’ and specialised nurse practioners’ outpatient clinic notes were analysed. Patient demographics (age, comorbidity, use of anticoagulant drugs, smoking, tumour stage, type of operation, blood loss and comorbidity) were noted. The Charlston comorbidity index was used to assess comorbidity [12]. Infection was defined as any wound appearance that required antibiotic treatment or surgery to evacuate infected seroma or abscess. Seroma was defined as a fluid collection as evidenced via palpation or clear serous fluid that was aspirated. Seroma aspirations were counted as registered in the patients’ charts.

#### Statistics

Continuous variables are presented as means with standard deviations or as medians with first and third quartiles as appropriate; categorical variables are presented as percentages. Continuous variables were compared between study groups with Student *t* tests or Mann-Whitney *U* tests as appropriate. Categorical variables were compared between study groups with chi-squared tests or Fisher exact tests as appropriate. The risk of complications according to study group was estimated using simple logistic regression. Potential confounding by relevant baseline characteristics was corrected for using multiple logistic regressions. The clinically relevant interaction between study group and operation type was assessed with the significance of the change in −2 log likelihood after inclusion of the interaction term in the logistic regression models. In case of significant interaction, simple effects were reported by stratified cross-tables. *p* < 0.05 was considered evidence of statistical significance.

## Results

In total, 835 breast cancer surgeries were performed in both 10-month intervals.

One hundred and eighty patients were included: 92 in the FF group and 88 in the HC group. Patient demographics (age, comorbidity, use of anticoagulant drugs, smoking, tumour stage, type of operation, blood loss and comorbidity) were not significantly different (Table 1).

**Table 1:** Patient characteristics and surgical aspects

	Flap fixation	Historical control	*p*
	*N* = 92	*N* = 88	
Age (years)	67 (±13)	71 (±11)	0.07
Comorbidity	2.8 (±1.4)	3.0 (±1.1)	0.45
Anticoagulant drugs (yes)	25.0 %	34.1 %	0.18
Smoking (yes)	22.8 %	23.9 %	0.82
Tumour stage			0.34
T1-2N0	44.6 %	50.0 %	
T1-2N+	25.0 %	19.3 %	
T3	14.1 %	9.1 %	
T4	3.3 %	9.1 %	
DCIS	13.0 %	12.5 %	
Operation			0.12
MRM	37.0 %	36.4 %	
Mastectomy	6.5 %	15.9 %	
Mastectomy + sentinel node	56.5 %	47.7 %	
Blood loss			0.11
0 mL	48.9 %	36.4 %	
0–50 mL	18.5 %	17.0 %	
50–100 mL	17.4 %	13.6 %	
100–150 mL	3.3 %	2.3 %	
>150 mL	11.9 %	30.7 %	

Data are presented as means with standard deviations or as percentages. All patients were evaluated

In the group that underwent flap fixation, 33/92 (35.9 %) patients developed a seroma, compared to 52/88 (59.1 %) patients in the HC group (*p* = 0.002). Seroma aspiration was performed in 14/92 (15.2 %) patients in the FF group as opposed to 38/88 (43.2 %) patients in the historical control group (*p* < 0.001). The number of seroma aspirations per patient that underwent seroma aspiration was also significantly reduced in the group undergoing flap fixation (*p* < 0.001). There was no significant difference in patients developing SSIs (12.0 % in the FF group and 17.0 % in the HC group, *p* = 0.33). These results are shown in Table 2.

**Table 2:** Postoperative complications

	Flap fixation	Historical control	*p*
	*N* = 92	*N* = 88	
Any complication	39.1 %	63.6 %	0.001
Seroma	35.9 %	59.1 %	0.002
Seroma requiring aspiration	15.2 %	43.2 %	<0.001
Number of aspirations	0 (0–0)	0 (0–1)	<0.001
Hematoma	5.4 %	1.1 %	0.21
SSI	12.0 %	17.0 %	0.33
Pneumothorax	0.0 %	1.1 %	0.49

Multivariate analysis revealed a trend in smokers to develop seroma (*p* = 0.05), as shown in Table 3.

**Table 3:** Multiple logistic regression analysis

	Seroma		Seroma requiring aspiration	
	Odds ratio (95 % CI)	*p*	Odds ratio (95 % CI)	*p*
Flap fixation (yes)	0.41 (0.22–0.79)	0.008	0.29 (0.14–0.60)	0.001
Age (years)	1.02 (0.99–1.05)	0.33	1.02 (0.99–1.06)	0.24
Operation		0.20		0.96
Mastectomy (reference)	–		–	
Mastectomy and sentinel node	1.13 (0.39–3.34)		0.96 (0.31–2.92)	
Modified radical mastectomy	2.09 (0.66–6.64)		1.07 (0.33–3.55)	
Comorbidity	1.06 (0.77–1.47)	0.72	1.25 (0.89–1.76)	0.20
Anticoagulant drugs (yes)	0.85 (0.37–1.96)	0.71	0.79 (0.32–1.96)	0.61
Smoking (yes)	2.16 (0.99–4.70)	0.05	0.71 (0.30–1.69)	0.44

When analysing the effect of flap fixation on seroma formation stratified by operation type, no effect was seen in the group undergoing mastectomy (*p* = 0.16 for seroma formation and *p* = 0.35 for seroma aspiration). In the group undergoing mastectomy and a sentinel node procedure, seroma formation was significantly reduced (FF 25.0 %, HC 61.9 %, *p* < 0.001) as well as seroma aspirations (FF 13.5 %, HC 45.2 %, *p* = 0.001). In patients undergoing MRM, there were no differences in seroma formation (55.9 % in the FF group and 56.2 % in the HC group); however, there was a statistical difference in the seroma aspirations in this group. Fewer patients required seroma aspiration in the group undergoing flap fixation (flap fixation 17.6 %, historical control 40.6 %, *p* = 0.04). These results are shown in Table 4.

**Table 4:** Effects of flap fixation on seroma formation stratified by operation type

Seroma (%)			
	Historical control	Flap fixation	*p*
Mastectomy	8/14 (57.1 %)	1/6 (16.7 %)	0.16
Mastectomy and sentinel node	26/42 (61.9 %)	13/52 (25.0 %)	<0.001
Modified radical mastectomy	18/32 (56.2 %)	19/34 (55.9 %)	0.98
Seroma requiring aspiration (%)			
	Historical control	Flap fixation	*p*
Mastectomy	6/14 (42.9 %)	1/6 (16.7 %)	0.35
Mastectomy and sentinel node	19/42 (45.2 %)	7/52 (13.5 %)	0.001
Modified radical mastectomy	13/32 (40.6 %)	6/34 (17.6 %)	0.04

## Discussion

This study demonstrates that reduction of the dead space after mastectomy using flap fixation reduces seroma formation and seroma aspirations. For many decades, breast surgeons have used closed suction drainage to reduce dead space. However, seroma formation and its sequelae continued to cause postoperative problems in these patients, proving that wound drainage is insufficient in combating seroma. Flap fixation combined with low suction drainage significantly reduces seroma formation and the need for seroma aspiration after mastectomy.

The key to reducing seroma formation seems to partly lie in the obliteration of dead space. However, the techniques used to achieve this goal are subject of much controversy and debate [6]. In a randomised controlled study, it was difficult to elucidate whether reducing the dead space or ligation of lymphatics or a combination of both were responsible for reduction of seroma formation [13]. The extent of the dissection plane seems to be an important factor in seroma formation, and therefore, obliteration of dead space in patients undergoing mastectomy or modified radical mastectomy seems to be pivotal. Pressure garments or compression bandages are not effective in combating seroma; however, quilting of the skin flaps or skin flap fixation seems to be much more effective [10, 14].

When analysing the effect of flap fixation on seroma formation stratified by operation type, no significant effect was seen in seroma formation or seroma aspiration in the group undergoing mastectomy alone (seroma formation *p* = 0.16, seroma aspiration *p* = 0.35). The low number of patients in this subgroup could explain the non-significance, as a clear trend is visible. If this group had been larger (*n* = 20), the difference possibly might have been statistically significant.

In the group undergoing MRM, flap fixation was less effective on seroma formation, although there were fewer seroma aspirations. In this group of patients, seroma formation could be more pronounced due to the axillary lymph node dissection, and therefore, there might be a relative under treatment of the axilla when evaluating seroma prophylaxis. Several studies have been performed to assess the effect of sealing devices on seroma formation in axillary dissection [7]. The use of the harmonic scalpel has been shown to reduce the magnitude of seromas in the axilla [15]. In another prospective randomised controlled trial performed by Cortadellas et al., the use of an electrothermal bipolar vessel sealing system (LigaSure) in axillary dissection was assessed. The mean number of postsurgical seroma aspirations and the amount of seroma fluid drained were lower in the LigaSure group; however, they were not statistically significant [16].

No significant differences in SSIs were seen in this study. This could be due to the fact that all patients were treated with prophylactic antibiotics and strict operating room discipline was in place. This entailed limiting door openings in theatre and thus limiting movements of theatre staff during procedures. A difference of 5 % in infection rates between both groups was too small to achieve statistical significance. The reported rates of SSIs after breast operations range dramatically from 0.8 to 26 % in the literature. One possible factor accounting for such wide-ranging SSI rates is the use of different definitions of SSIs [17].

Cosmesis and shoulder function on the ipsilateral side were not evaluated in this study. One patient in the flap fixation group suffered from a diminished shoulder function postoperatively. It was unclear whether there was full range of motion preoperatively. Another patient in the flap fixation group appeared to develop skin dimpling on the chest wall 6 months after surgery. No studies to date have been published regarding cosmesis, shoulder function and patient satisfaction with long-term follow up after flap fixation. This should be addressed in a prospective trial.

The main limitations of this study are related to the retrospective nature. Being a retrospective study, indications for seroma aspiration had not been defined beforehand. This might be a potential confounder in this study. However, this is the first study to evaluate seroma aspiration in mastectomy patients treated with flap fixation and low suction wound drainage. Assessing the presence of seroma is difficult due to the subjective nature of this procedure. How does one objectively measure the presence of seroma? Maybe the only true measure for seroma is seroma aspiration. There were no major policy changes in the treatment of seroma in both time intervals, but bias is of course possible. There does seem to be an increasing surgical tendency towards watchful waiting when treating postoperative wound seroma.

Seroma aspiration is clinically relevant to our group of patients, and it is an important cause of patient discomfort [2]. Seroma leads to prolonged hospital stay, a higher rate of infections and therefore delayed administration of adjuvant treatment [18]. Patients undergo more frequent outpatient clinic visits, a higher rate of surgical reinterventions and patients may have a worse cosmetic outcome [10]. Finally, the cost of medical care is higher in the group of patients suffering from seroma and its sequelae [19].

The strength of this study lies in the proven efficacy of flap fixation after mastectomy in 180 patients in two large teaching hospitals. The most important outcome is diminished seroma aspirations in the group having undergone flap fixation when compared to patients with only low suction drainage postoperatively. This in itself should lead to a further reduction of patient discomfort.

## Conclusions

Flap fixation is a surgical technique that reduces the dead space in patients undergoing mastectomy for invasive breast cancer or DCIS. It appears to reduce the occurrence of seroma and the need for seroma aspirations. A prospective trial can further evaluate the effect of flap fixation, including long-term outcome measures such as cosmesis, shoulder function and patient satisfaction.
